# Data on agricultural and nonagricultural land use in peri-urban and rural area

**DOI:** 10.1016/j.dib.2019.103804

**Published:** 2019-03-01

**Authors:** Mohammad Rondhi, Pravitasari Anjar Pratiwi, Vivi Trisna Handini, Aryo Fajar Sunartomo, Subhan Arif Budiman

**Affiliations:** a Departement of Agribusiness, Faculty of Agriculture, University of Jember, Indonesia; b Departement of Agricultural Extension, Faculty of Agriculture, University of Jember, Indonesia; c Departement of Soil Science, Faculty of Agriculture, University of Jember, Indonesia

**Keywords:** Peri-urban area, Rural area, Land use characteristics

## Abstract

The data in this article describes the land use characteristics at peri-urban and rural areas, on Jember District, in the Province of East Java, Indonesia. The types of land use covered in the data are agricultural and residential land. The data was a result of a research collaboration between the Department of Agribusiness, Department of Soil Science, and the Department of Agricultural Extension in the University of Jember. The general purpose of the data collection was to compare the characteristics of different land use in the peri-urban and rural area. The data has been compiled to investigate the economic rent of varying land use in peri-urban and rural areas to explain the dynamic of farmland conversion, and to investigate the farmland distribution among farmer in the peri-urban area. The data contains technical and socio-economic aspects of land use in peri-urban and rural areas. The data were collected through structured interviews with farmers and homeowners in each area. A total of 200 interviews were conducted to 100 farmers and homeowners. The location of each respondent was recorded with the location-marking feature of the GPS to represent the distribution of samples. The tracking feature of the GPS was used to locate the physical infrastructure such as irrigation canal, road, and market. In total, the data contained 29 variables and attached as the supplementary material of this data article.

**Table:** Specifications table

Subject area	*Agriculture, Geography, Economics*
More specific subject area	*Agricultural economics*
Type of data	*Table and Figure*
How data was acquired	*Structured interviews with farmers and homeowners using questionnaire; geographic location of each house and farmland of the respondents determined using handheld GPS.*
Data format	*Raw*
Experimental factors	*The data was undergone correction from entry errors, nonresponse, and inaccurate GPS coordinates*
Experimental features	*50 farmers and 50 homeowners were randomly selected in each area*
Data location	*Kepanjen village, subdistrict of Gumukmas (rural) and Antirogo village, subdistrict of Sumbersari (peri-urban) in the District of Jember, Province of East Java, Indonesia*
Data accessibility	*The data was attached to this data article as Supplementary Material*
Related research article	1. M. Rondhi, P.A. Pratiwi, V.T. Handini, A.F. Sunartomo, S.A. Budiman, **Agricultural land conversion, land economic value, and sustainable agriculture: A case study in East Java, Indonesia**[1] 2. P.A. Pratiwi, M. Rondhi, **Distribusi Kepemilikanahan Pertanian Dan Pendapatan Usahatani Di Wilayah Perkotaan Kabupaten Jember**[2]

**Table:** 

**Value of the data** • The data can be used to comparefarmingpractice in rural and peri-urban areas. • The data can be used to investigate the competition between agricultural and nonagricultural land use in the rural and peri-urban areas. • The data can be used to compare the pesticide use behavior between rural and peri-urban farmer. • The data can be used to compare the farming practice between food and nonfood crops. • The data can be used to measure the economic rent of different land use in the rural and peri-urban area.

## 1 Data

**Fig. 1:**
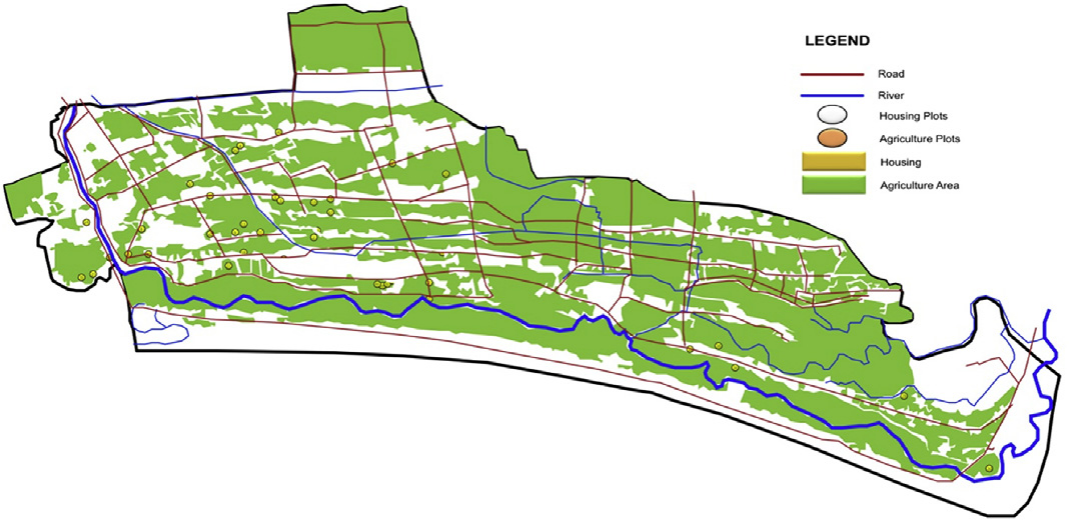
The distribution of the sampled farmland in the rural area.

**Fig. 2:**
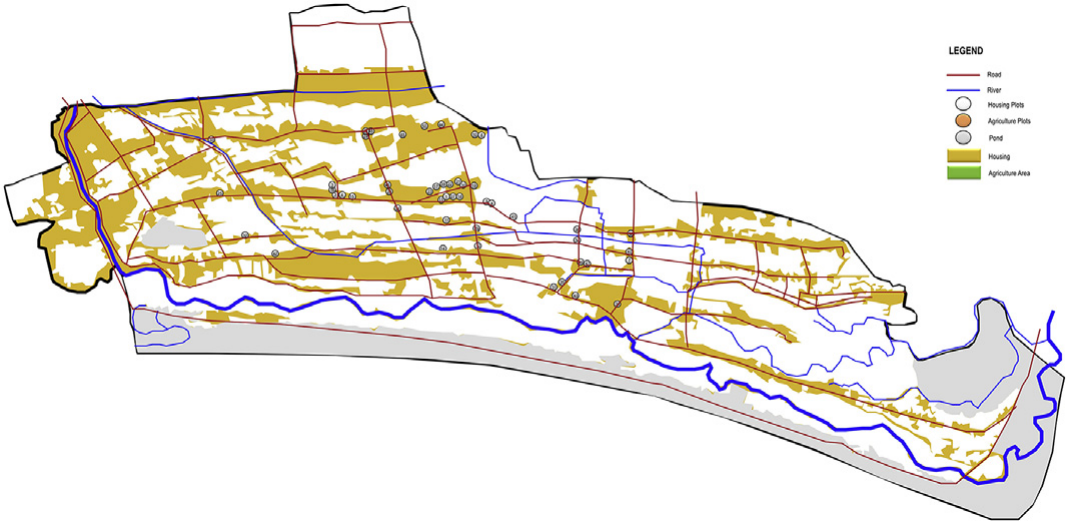
The distribution of the sampled house in the rural area.

**Fig. 3:**
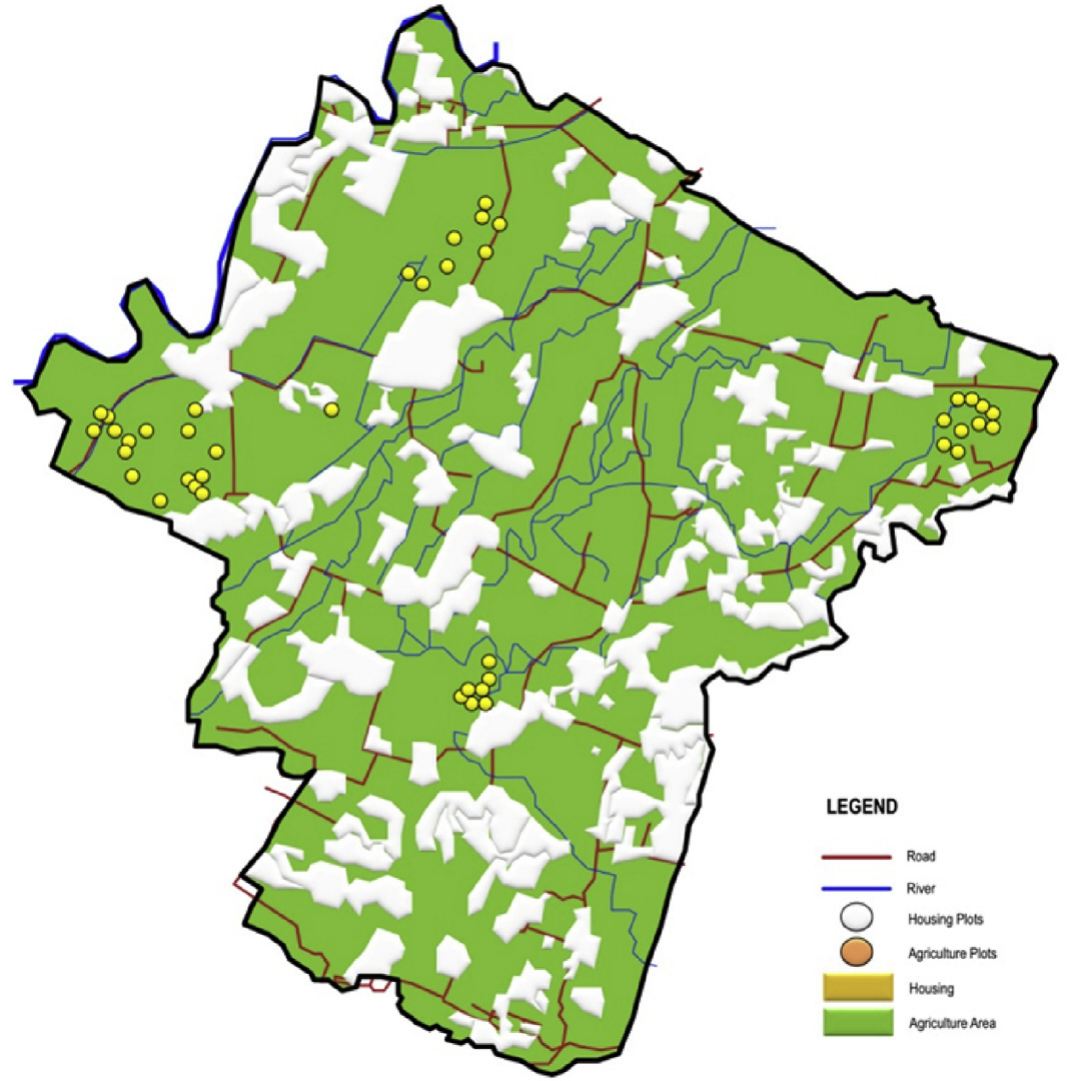
The distribution of the sampled farmland in the peri-urban area.

**Fig. 4:**
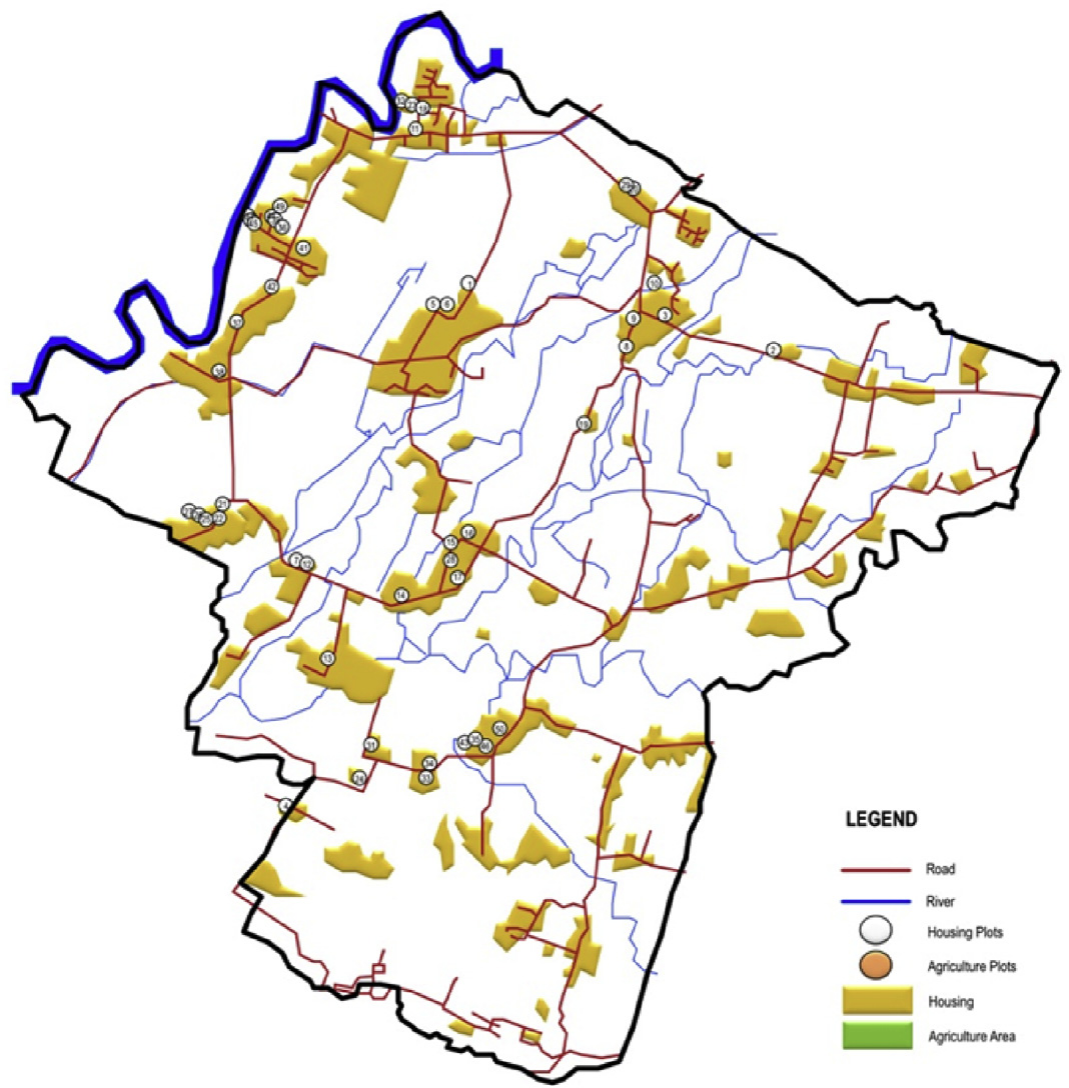
The distribution of the sampled house in the peri-urban area.

The data contains information on technical and socio-economic aspects of farmland and housing in the rural and peri-urban areas. The exact location of each rural farmland (Fig. 1), rural house (Fig. 2), peri-urban farmland (Fig. 3), and peri-urban house (Fig. 4) were determined using GPS. The variables representing technical and socio-economic aspects were collected through interviews with farmers and homeowners. The description of each variable, the unit of measurement, the nature of data, and the source from which the data were obtained are presented in Table 1. In addition, the data regarding the general conditions of each village were obtained from the village's official profile.

**Table 1:** The variables in the data.

Subjects	Variables	Descriptions	Measure	Unit	Source
Agriculture	age	Farmers age in the interview	Scale	yr	Interview
	education	The latest formal education certificate (1 = elementary education; 2 = junior high school; 3 = senior high school; 4 = higher education)	nominal		Interview
	landtype^a^	The type of land cultivated by farmers (1 = wetland; 2 = moorland; 3 = swampland)	nominal		Interview
	crop	The type of crop planted on a particular season			
	production	The produce of the crop planted on a particular season	Scale	kg	Interview
	harvest_price	The price of the produced crop at harvest	Scale	rp kg^−1^	Interview
	lab_cost	The cost of labor for a particular season		rp	Interview
	ferti_cost	The total cost of fertilizer for a particular season	Scale	rp	Interview
	pest_cost	The total cost of pesticides for a particular season	Scale	rp	Interview
	irri_cost	The cost of irrigation water for a particular season	Scale	rp	Interview
	mech_cost^b^	The cost of agricultural machinery services for a particular season	Scale	rp	Interview
	seed_cost	The total cost of seed for a particular season	Scale	rp	Interview
	irri_infra	The existence of irrigation infrastructure (1 = exists; 2 = doesnt exists)	Nominal		Interview
	land_loc	The coordinate (latitude and longitude) of farmer's land	Scale		GPS
Housing	age	The age of the homeowner at the time of the interview	Scale	yr	Interview
	occupation	The main occupation of homeowners			
	education	The latest formal education certificate (1 = elementary education; 2 = junior high school; 3 = senior high school; 4 = higher education)	nominal		Interview
	rent_fee	The rental fee of the house has the house been rented	Scale	rp yr^−1^	Interview
	water_cost	The cost of monthly water service	Scale	rp	Interview
	electric_cost	The monthly cost of electricity service	Scale	rp	Interview
	maint_cost	The monthly cost of house maintenance	Scale	rp	Interview
	bathroom^c^	Number of the bathroom in the house	Scale	unit	Interview
	trans_access	The ease of transportation access to the house (1 = easy; 2 = difficult)	Nominal		Interview
	envir_pollution^d^	Homeowner's perception of environmental pollution in the environment (1 = not polluted; 2 = polluted)	Nominal		Interview
	house_loc	The coordinate (latitude and longitude) of the house	Scale		GPS
Village	landuse_distrib	The distribution of land for various uses in each village.	Scale	ha	[3], [4]
	vill_area	The area of the entire village	Scale	km sq	
	vill_population	The resident population of each the village	Scale	person	
	vill_agriculture	The type of cultivated crop			

a The variable land type only available for rural farmers.

b The variable agricultural machinery service cost only available for rural farmers.

c The variable of number of the bathroom only available for rural house.

d The variable of environmental pollution only available for housing in the peri-urban area.

The data is provided in a Microsoft Excel format consisting of six sheets. The first sheet contains information on rural agriculture; the second contains variable on rural housing. The information on peri-urban agriculture and housing are contained in the third and fourth sheets. For each land use (agricultural and housing), we used the same variables regardless of the area. Finally, the general conditions of each village are presented in the fifth and sixth sheets.

## 2 Experimental design, materials, and methods

The sampling determination was conducted in two stages. The first stage was aimed to determine the population of farmers and homeowners in the rural and peri-urban area. The population of farmers in the rural area is 783 farmers, while there are 1056 farmers in the peri-urban area. The population homeowners in the rural and peri-urban area are 3011 and 3050 respectively. In the second stages, 50 farmers and 50 homeowners in each village were randomly selected as the final sample.

The data collection was conducted in two stages. In the first stage, we collect data on the technical and socio-economic characteristics of farmers and homeowners. The data was collected through personal interviews to farmer and homeowner using a structured questionnaire by a trained enumerator. In the second stage, the location of each house and farmland, as well as irrigation canal, road and market were geolocated using GPS by a different team of the surveyor.
